# Molecular characteristics of multiple primary pulmonary nodules under a three-dimensional reconstruction model and relevant multi-omics analyses: a case report

**DOI:** 10.3389/fonc.2023.1064475

**Published:** 2023-05-02

**Authors:** Zhilin Luo, Yajie Xiao, Chengwen Luo, Liping Zhang, Runquan Zhou, Zhikun Zhao, Chao Sun, Dongfang Wu, Tianhu Wang

**Affiliations:** ^1^ Department of Thoracic Surgery, The Third Affiliated Hospital of Chongqing Medical University, Chongqing, China; ^2^ Department of Medicine, YuceBio Technology Co. Ltd., Shenzhen, China

**Keywords:** multiple primary lung cancer, multi-omics analyses, genomic profiling, tumor environment, three-dimensional (3D) reconstruction

## Abstract

**Background:**

In addition to CT images and pathological features, many other molecular characteristics remain unknown about multiple primary lung cancer (MPLC) from intrapulmonary metastatic lung cancer.

**Case presentation:**

In this study, we reported a patient with an early-stage MPLC with both adenocarcinoma *in situ* (AIS) subtype and minimally invasive adenocarcinoma (MIA) subtype. The patient was diagnosed with more than 10 nodules and underwent precise surgery assisted by three-dimensional (3D) reconstruction at the left upper lung lobe. Whole-exome sequencing (WES) and multiple immunohistochemistry (mIHC) were performed to reveal the genomic profiling and tumor microenvironments of multiple nodules in this patient with MPLC. Based on 3D reconstruction location information, we found that the genomic and pathological results of adjacent lymph nodes were quite different. On the other hand, PD-L1 expression and the proportion of infiltrating lymphocytes in tumor microenvironments were all at a low status and did not vary in adjacent lymph nodes. Additionally, maximum diameter and tumor mutational burden levels were found to be significantly associated with CD8+ T cell proportion (p<0.05). Besides, CD163+ macrophages and CD4+ T cell proportion were higher in MIA nodules than in AIS nodules (p<0.05). This patient reached a recurrence-free survival of 39 months.

**Conclusion:**

Generally, in addition to CT imaging and pathological results, genomic profiling and tumor microenvironments may facilitate identifying the potential molecular mechanisms and clinical outcomes in patients with early-stage MPLC.

## Introduction

In 2015, the World Health Organization (WHO) classified two new subtypes of lung adenocarcinoma: adenocarcinoma *in situ* (AIS) and minimally invasive adenocarcinoma (MIA) (1). Based on CT images, the AIS subtype of lung adenocarcinoma was defined as a small lesion (≤3 cm) consisting of a pure lepidic component. Whereas, the MIA type was classified as a small lesion (≤3 cm) consisting of a predominantly growing lepidic component together with some invasive small components (2).

These two subtypes of lung adenocarcinoma have been proven to have good long-term prognoses upon timely diagnosis and operation (3, 4). The 5-year recurrence-free survival (RFS) of AIS patients and MIA patients reaches nearly 100% after complete resection (5, 6). Morphologically, it is hypothesized that a gradual malignant progression can be generated from AIS to MIA. However, definite molecular mechanisms for this transformation remain unclear. Many current studies focused on early diagnosis characteristics from CT images (7–9), while only a few studies conducted comprehensive analyses for molecular profiles (10).

In this study, the patient was diagnosed with multiple primary lung cancer (MPLC) with more than 10 nodules of both AIS and MIA subtypes and subsequently underwent precise lobectomy assisted by three-dimensional (3D) reconstruction. Additionally, we compared molecular characteristics between AIS and MIA subtypes in terms of the CT images, 3D locations, genomic profiles, and tumor microenvironments in order to provide potential clinical evidence for identifying the molecular mechanisms in early lung cancer.

## Case presentation

**Figure 1:**
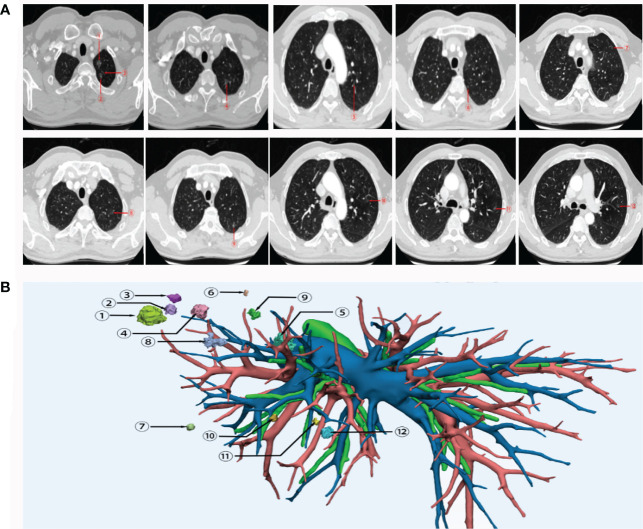
CT images **(A)** and 3D reconstruction **(B)** of this patient with early-stage MPLC. Nodule 1, nodule 3, nodule 5, nodule 6 and nodule 9 were identified as MIA. Nodule 4, nodule 12, and nodule 11 were identified as AIS. No cancer cells were found in nodule 2, nodule 7, nodule 8, and nodule 10. MPLC, multiple primary lung cancer; AIS, adenocarcinoma in situ; MIA, minimally invasive adenocarcinoma.

A 55-year-old man who was a mild smoker, and had no medical or family history, had his annual physical examination in January 2019 and was found with a small nodule (about 1cm) in the upper lobe of the left lung. Three months later, he was observed with multiple small ground-glass nodules (about 1cm) in the upper lobe of the left lung. After oral moxifloxacin anti-inflammatory treatment, the CT images of the patient did not improve. On the contrary, in July 2019, his CT images revealed multiple enlarged nodules in the upper lobe of the left lung and he was admitted to our hospital for further treatment (**Figure 1A**). Then, the patient was diagnosed with more than 10 nodules and we used a three-dimensional (3D) reconstruction model to evaluate the accurate location of these nodules and their relevant anatomical structures (**Figure 1B**). Subsequently, we conducted a precise operation of the left upper lung lobe, including the posterior end of the tip of the upper lobe and the anterior segment of the upper lobe. Pathological results showed that nodule 1, nodule 3, nodule 5, nodule 6, and nodule 9 were identified as MIA while nodule 4, nodule 12, and A subsegment (nodule 11) were identified as AIS. No cancer cells were found in nodule 2, nodule 7, nodule 8, and nodule 10. Based on the latest follow-up in October 2022, the patient was still in good condition and no evidence of disease recurrence was found. Thereby this patient reached a 39-month RFS.

**Figure 2:**
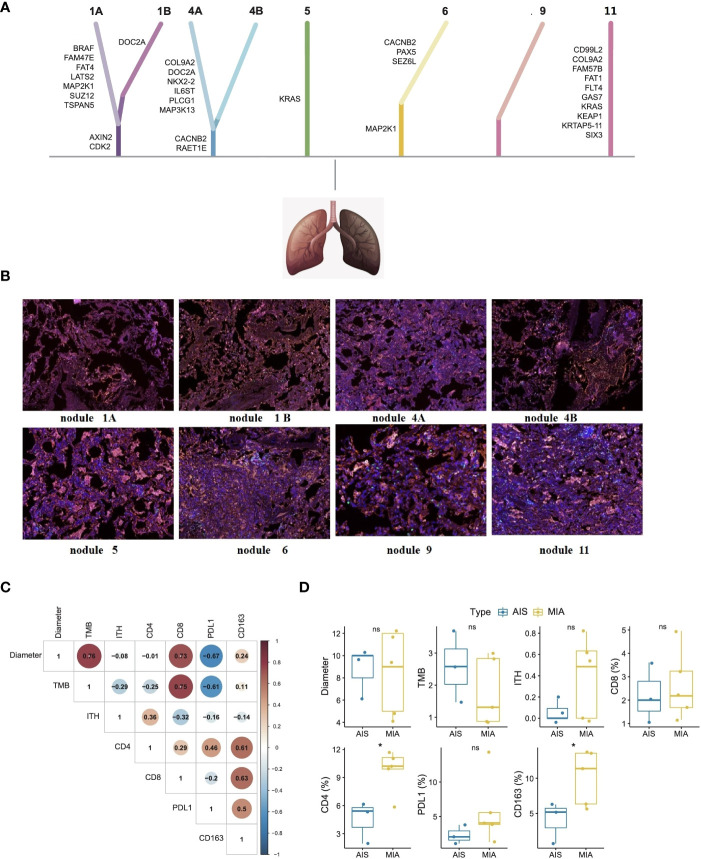
Whole-exome sequencing **(A)**, mIHC analysis **(B)**, biomarker correlations **(C)**, and subtype differences **(D)** of each nodule in this patient with early-stage MPLC. Whole-exome sequencing showed that there were almost no collective mutations among all nodules. mIHC analysis showed similarly low positive levels of CD4 + T cells (yellow color), CD8 + T cells (green color), CD163+ macrophages (purple color), and PD-L1 expression (red color) in tumor microenvironments of each nodule. Fisher’s exact test and Wilcox test were used to analyze the correlation or differences between different groups. *p<0.05 was considered as statistically significant. MPLC, multiple primary lung cancer; AIS, adenocarcinoma in situ; MIA, minimally invasive adenocarcinoma. ns, not statistically significant.

In this current case, whole-exome sequencing (WES) (**Figure 2A**) and multiple immunohistochemistry (mIHC) (**Figure 2B**) were performed to reveal the genomic profiling and tumor microenvironments of multiple nodules, including nodule1, nodule 4, nodule 5, nodule 6, nodule 9, and A subsegment (nodule 11). Based on WES results, all the nodules were at low tumor mutational burden (TMB) ranging from 0.46-3.68 muts/mb, low tumor neoantigen burden (TNB) ranging from 0.03-1.19 neos/mb, and low microsatellite instability (MSI) ranging from1.21-19.08%. The nodules did not present any loss at human leukocyte antigen (HLA). As shown in **Figure 2A**, *KRAS* mutations were observed at nodule 5, mutations at those genes, including *MAP2K1, SEZ6L, PAX5* and *CACNB2*, were observed at nodule 6, no mutations were observed at nodule 9, and mutations at those genes, including *SIX3, KRTAP5-11, KEAP1, KRAS, GAS7, FLT4, FAT1, FAM57B, COL9A2, CD99L2*, were observed at A subsegment (nodule 11). Except for KRAS in nodule 5 and A subsegment (nodule 11), there were almost no collective mutations among all nodules. By associating with 3D locations, we found that the genomic and pathological results of adjacent lymph nodes were quite different, implying MPLC instead of intrapulmonary metastatic lung cancer (IPM). To illustrate the intratumor heterogeneity, we used opposite sections of a wax block for nodule 1 and nodule 4 to perform WES and mIHC analysis. Mutations at those genes, including *AXIN2, CDK2, TSPAN5, SUZ12, MAP2K1, LATS2, FAT4, FAM47E*, and *BRAF*, were observed at nodule 1A, while mutation at those genes, including *CDK2, AXIN2*, and *DOC2A*, were observed at nodule 1B. Mutations at those genes, including *RAET1E, CACNB2, MAP3K13, PLCG1, IL6ST, NKX2-2, DOC2A, COL9A2*, were observed at nodule 4A while mutation at those genes, including *CACNB2* and *RAET1E*, were observed at nodule 4B. Overall, only a few mutations were simultaneously found at opposite sections of the wax block in either nodule 1 or nodule 4 (*CDK2* and *AXIN2* mutations in nodule 1A and nodule 1B; *CACNB2* and *RAET1E* mutations in nodule 4A and nodule 4B).

As seen in **Figure 2B**, mIHC analysis showed similarly low positive levels of CD4 + T cells (less than 17%), CD8 + T cells (less than 5%), CD163+ macrophages (less than 15%), and PD-L1 expression (less than 15%) in tumor microenvironments of each nodule. As shown in **Figure 2C**, maximum diameter and TMB levels were found to be significantly associated with CD8+ T cell proportion (p<0.05). Unfortunately, there was no definite correlation in PD-L1 level, ITH level, CD4+ T cells proportion, and CD163+ macrophage proportion. Comparing the relevant results of MIA and AIS nodules in **Figure 2D**, CD163+ macrophages and CD4+ T cell proportions were higher in MIA nodules than in the AIS nodules (p<0.05).

## Discussion

Beyond CT imaging and pathological finding, many other molecular characteristics remain unknown about MPLC, with both AIS and MIA. Due to the patient being diagnosed with more than 10 nodules, he underwent a precise lobectomy assisted by 3D reconstruction. Combined with 3D location information, the genomic and pathological results of adjacent lymph nodes were quite different, implying MPLC instead of IPM. Besides, this patient with early-stage MPLC showed similarly low positive levels of predictive biomarkers such as TMB, TNB, MSI, and HLA loss of heterozygosity for each nodule. In addition, PD-L1 expressions as well as the proportion of infiltrating lymphocytes in tumor microenvironments were low positive in both AIS and MIA and did not vary significantly in adjacent lymph nodes. Additionally, maximum diameter and TMB level were found to be significantly associated with CD8+ T cell proportion. Besides, CD163+ macrophages and CD4+ T cell proportion were higher in MIA nodules than in AIS nodules.

Comprehensive multi-omic analysis of multidisciplinary factors leaves space for clarifying potential molecular mechanisms. As is known, genomic profiling could identify the clonality status of multiple lesions. The TRACERx project revealed the tumor evolution in patients with early-stage NSCLC and suggested that driver gene mutations in EGFR, MET, BRAF, and TP53 are the most common clonal driver mutations in lung adenocarcinoma (11). Some retrospective studies also showed that EGFR mutations were more common in MIA than in AIS (8, 12, 13). WES results of multiple nodules in this patient only revealed BRAF mutation in nodule 1, and no other common mutations were found. Taking advantage of the 3D reconstruction technique, we found that the genomic and pathological results of adjacent lymph nodes were quite different, indicating MPLC instead of IPM. Compared with other previous studies that focused only on genomic analysis or CT findings, we combined multi-dimensional information to stereoscopically display the genomic and pathological characteristics of MPLC for distinguishing it from IPM.

Besides, the tumor microenvironment plays a crucial role in cancer evolution at all stages of NSCLC, including development, invasion, and metastasis (14, 15). The dynamic balance of anti- and pro-inflammatory immune cells can drive the immunoediting process and relative immune host response. Even after complete surgical treatment, the prognosis of NSCLC is variable and nearly half of all surgical patients develop post-surgery recurrence (16). More and more evidence suggests that adjuvant systemic therapies, such as immunotherapy, can lead to additional benefits and reduce the risk of recurrence for these early-stage surgical patients (16). Therefore, molecular biomarkers for predicting NSCLC post-surgery prognosis are urgently needed. In early-stage lung cancer, the presence of CD8+ T cells, CD4+ T cells, and CD20+ B cells were positive predictors of decreasing risks of death (17). In this study, we used multiple immunohistochemistry (mIHC) of CD4 + T cells, CD8 + T cells, CD163+ macrophages, and PD-L1 expression for analysis of immune characteristics of tumor microenvironments. The proportion of infiltrating lymphocytes of CD4 + T cells, CD8 + T cells, CD163+ macrophages, and PD-L1 expression was at a low positive level in this patient, suggesting that the immunoediting process and relative immune host response had partially initiated. In addition, higher maximum diameter and higher TMB levels were found to be significantly associated with higher CD8+ T cell proportion (p<0.05), indicating that there migtht be a stronger immune host response in lesions with larger sizes. In addition, infiltrating CD163+ macrophages and CD4+ T cell proportion were higher in MIA than in AIS. Other similar studies showed that CD4+ infiltrating T cell was higher in MIA than in AIS, implying tumor invasion (18, 19). This seemed consistent with the WHO definition of the two subtypes. Contrary to genomic features, we found that the immune characteristics of adjacent lymph nodes are not very different based on the 3D reconstruction model. After undergoing precise lobectomy assisted by 3D reconstruction, the patient did not receive any adjuvant systemic therapies and reached a 39-month RFS. This study might suggest that low positive expression of multiple biomarkers in tumor microenvironments might be useful predictors of long-term prognosis of complete surgical resection without adjuvant systemic therapy.

In summary, in addition to CT imaging and pathological results, genomic profiling and tumor microenvironments may help with clarifying the potential molecular mechanism in patients with early-stage MPLC. Moreover, multiple biomarkers in tumor microenvironments may be developed as prognosis predictors for long-term outcomes of early lung cancer patients after surgery.

## Data availability statement

The original contributions presented in the study are included in the article/**Supplementary Material**. Further inquiries can be directed to the corresponding author.

## Ethics statement

Written informed consent was obtained from the individual(s) for the publication of any potentially identifiable images or data included in this article.

## Author contributions

TW: supervision. ZL and YX: conceptualization and writing - original draft preparation. CL, LZ, and RZ: methodology and writing - review and editing. YX, ZZ, and DW: formal analysis. CS: editing. All authors contributed to the article and approved the submitted version.
