# Supplementary material for: Molecular characteristics of multiple primary pulmonary nodules under a three-dimensional reconstruction model and relevant multi-omics analyses: a case report
Source: Front Oncol. 2023 May 2;13:1064475. doi: 10.3389/fonc.2023.1064475 (PMC10185815; doi:10.3389/fonc.2023.1064475)
Supplement: Supplementary file 1 [file Table_1.doc]

Supplementary Table 1 Detailed characteristics of multiple primary nodes in this early-stage lung cancer patient

| **No** | **1A** | **1B** | **4A** | **4B** | **3** | **5** | **6** | **9** | **11**  **(A sub )** |
| --- | --- | --- | --- | --- | --- | --- | --- | --- | --- |
| **Subtype** | MIA | MIA | AIS | AIS | MIA | MIA | MIA | MIA | AIS |
| **Size(mm)** | 12 x 9 | 12 x 9 | 10 x 9 | 10 x 9 | 6 x 3 | 3 x 4 | 7 x 9 | 4 x 5 | 5 x 6 |
| **TMB (Mut/Mb)** | 3 | 2.83 | 2.58 | 3.68 | 4.37 | 0.87 | 0.84 | 1.31 | 1.47 |
| **TNB (Neo/Mb)** | 0.03 | 0.44 | 0.07 | 0.66 | 1.16 | 0.19 | 0.09 | 0.37 | 0.5 |
| **MSI (%)** | 14.38 | 2.38 | 19.08 | 0.61 | 2.99 | 2.38 | 3.01 | 1.21 | 3.09 |
| **ITH** | 0 | 0.49 | 0 | 0.19 | 0.33 | 0 | 0.63 | 0.82 | 0 |
| **HLA LOH** | negative | negative | negative | negative | negative | negative | negative | negative | negative |
| **Tumor cell (%)** | 40% | 10% | 40% | 5% | 25% | 20% | 25% | 10% | 30% |
| **CD4+** | 11.72% | 5.88% | 5.42% | 6.15% | 16.63% | 9.91% | 11.14% | 10.22% | 1.94% |
| **CD8+** | 4.96% | 3.24% | 2.00% | 3.61% | 1.07% | 2.18% | 1.68% | 1.16% | 1.06% |
| **PD-L1+** | 3.84% | 1.31% | 3.78% | 1.09% | 6.03% | 14.32% | 4.06% | 5.57% | 2.05% |
| **CD163+** | 13.56% | 11.38% | 6.27% | 5.18% | 14.72% | 13.70% | 6.34% | 5.64% | 0.70% |
| **Pan-Keratin+** | 15.73% | 13.91% | 6.91% | 10.20% | 8.55% | 10.37% | 4.25% | 0.84% | 4.05% |

*TMB*: tumor mutation burden (*Mut/Mb* mutations per megabase); *TNB:* tumor neoantigen burden (*Neo/Mb* neoantigens per megabase); *HLA* : human leukocyte antigen; *LOH:* loss of heterogeneity;
